# Serum levels of leptin, ghrelin putative peptide YY-3 in patients with fetal alcohol spectrum disorders

**DOI:** 10.1038/s41598-024-66052-7

**Published:** 2024-06-28

**Authors:** Rafał Podgórski, Sabina Galiniak, Artur Mazur, Agnieszka Domin, Dominika Podgórska

**Affiliations:** 1 Department of Biochemistry, Institute of Medical Sciences, Medical College of Rzeszow University, Warzywna 1a, 35-310 Rzeszow, Poland; 2 Department of Pediatric, Institute of Medical Sciences, Medical College of Rzeszow University, 35-310 Rzeszow, Poland; 3 Department of Rheumatology, Institute of Medical Sciences, Medical College of Rzeszow University, 35-310 Rzeszow, Poland

**Keywords:** Fetal alcohol spectrum disorders, FAS, Leptin, Ghrelin, Peptide YY-3, Endocrine system and metabolic diseases, Biochemistry

## Abstract

Fetal alcohol spectrum disorders (FASD) are a severe developmental condition resulting from exposure to alcohol during pregnancy. The aim of this study was to examine the concentrations of hormones involved in appetite regulation—ghrelin, leptin, and putative peptide YY-3 (PYY)—in the serum of individuals with FASD. Additionally, we investigated the relationship between these hormone levels and clinical indicators. We conducted an enzyme-linked immunosorbent assay on samples collected from 62 FASD patients and 23 individuals without the condition. Our results revealed a significant decrease in leptin levels among FASD patients compared to the control group (5.124 vs. 6.838 ng/mL, *p* = 0.002). We revealed no statistically significant differences in the levels of other hormones studied (ghrelin and PYY). Comparisons of hormone levels were also conducted in three subgroups: FAS, neurobehavioral disorders associated with prenatal alcohol exposure and FASD risk, as well as by sex. Assignment to FASD subgroups indicated changes only for leptin. Sex had no effect on the levels of hormones. Moreover, the levels of leptin showed a negative correlation with cortisol levels and a positive correlation with BMI and proopiomelanocortin. Alterations in appetite regulation can contribute to the improper development of children with FASD, which might be another factor that should be taken into consideration in the proper treatment of patients.

## Introduction

Determination of hormonal biomarkers is increasingly significant across various diseases, including fetal alcohol spectrum disorders (FASD), a debilitating developmental disorder caused by prenatal alcohol exposure. The most significant abnormalities are associated with neurological disorders that lead to deficits in cognition, executive function, memory, hearing, vision, motor skills, behavior, and social adaptation^1^. Others developmental defects resulting from alcohol exposure include growth deficiencies of developing organs and body systems^2^. FASD encompasses a broad syndrome among which entities with different variability in dysmorphic and neurological features can be distinguished, such as fetal alcohol syndrome (FAS), partial fetal alcohol syndrome, alcohol-related neurodevelopmental disorder, and alcohol-related birth defects. FAS is the most severe form of FASD which can occur with the chronic consumption of high doses of alcohol during pregnancy. The diagnosis of FASD is based on specific criteria, including assessment of neonatal and postnatal growth retardation, characteristic facial dysmorphology, and central nervous system disorders, encompassing developmental delays, neurological abnormalities, and intellectual impairment^3^. However, currently there are no globally unified diagnostic criteria or classification system for FASD, and the most widely used criteria are those of the Institute of Medicine, the Fetal Alcohol Spectrum Disorder 4-Digit Diagnostic Code, the Canadian guidelines, and the Centers for Disease Control and Prevention guidelines^4–7^. The harmful effects of ethyl alcohol on the developing fetus were established more than 50 years ago, yet it remains a significant social and public health issue^8^. Recent data indicate that the global prevalence of FASD among children and adolescents is estimated at 7.7‰, which makes FASD the most common cause of neurodevelopmental disorders worldwide^9–11^. Estimating the actual prevalence of FASD is challenging, as not all countries have comprehensive records. These numbers are likely underestimated, and active case ascertainment in schools has revealed a prevalence of FASD of approximately 3.6% in the USA and Italy, and even 13.6 to 20.9% in South Africa^12–15^. Despite increased awareness and numerous educational programs about the detrimental effects of alcohol, the problem persists and may even be worsening. Moreover, the highest rates of risky drinking occur in adolescents and young adults (20–24 years), the age group with the highest incidence of unplanned pregnancies. Statistically, one in every 13 pregnant women who consumed alcohol during pregnancy would deliver a child with FASD and 1 in 67 women is estimated to deliver a child with FAS, resulting in approximately 119,000 children born with FAS worldwide each year^9^. The safe and allowable amount of alcohol during pregnancy is not known, and complete abstinence is recommended to avoid the risk of developing FASD^16^. In addition to structural anomalies and growth deficits, FASD is associated with a wide spectrum of neurobehavioral abnormalities, which can indirectly contribute to, among other, malnutrition. Fetal and long-term growth restriction is a persistent characteristic of FAS, but individuals with milder forms of FASD (e.g., alcohol-related neurodevelopmental disorder, partial FAS) may have an increased long-term risk of overweight compared to unexposed individuals^17,18^. Furthermore, inappropriate eating patterns are prevalent in children with FASD and can contribute to their poor nutrition and growth restriction^19^.

The regulation of food intake involves a complex network of peripheral and central mechanisms, converging within the appetite centers of the hypothalamus.. These centers respond by coordinating the release of various neuropeptides that either stimulate or suppress appetite^20^. Ghrelin and leptin, in particular, are recognized as pivotal hormones in food intake regulation, with ghrelin typically stimulating appetite and leptin exerting an inhibitory effect^21^. Another anorexigenic hormone involved in appetite control is putative peptide YY-3 (PYY), which is produced peripherally by the gastrointestinal tract, including the ileum, colon, and rectum^22,23^.

The objectives of this study were twofold: first, to determine the levels of leptin, ghrelin, and putative peptide YY in the serum of children with FASD and compare them with those of a healthy control group; second, to assess the correlation between these hormones and the clinical parameters of the patients. To the best of our knowledge, this is the first study to describe the levels of these hormones in individuals affected by FASD.

## Materials and methods

### Study group

A single-center cross-sectional study was carried out in 62 patients with FASD aged 5 months to 16.5 years and 23 healthy controls. The affected individuals were categorized into three subgroups: FAS, ND-PAE (neurobehavioral disorder associated with prenatal alcohol exposure) and FASD risk. Diagnosis of FASD was based on the most recent Polish recommendations^24^. In respect of internationally adopted guidelines, the ND-PAE domain includes: partial fetal alcohol syndrome, and alcohol-related neurodevelopmental disorder^4–7^. Participants were included from the Department of Pediatrics, Pediatric Endocrinology and Diabetology and the Endocrinology Outpatient Clinic between March 2019 and January 2022. All procedures involving human participants adhered to the ethical standards of the institutional and/or national research committee and were in line with the Declaration of Helsinki of 1964 and its subsequent amendments or comparable ethical standards. Informed consent was obtained from all participants or, for those under 16 years of age, from a parent and/or legal guardian. The study protocol received approval from the Institutional Ethics Committee of the University of Rzeszow (16/02/2019).

### Blood sampling

Blood sampling was conducted in the morning between 8:00 and 10:00 am, following an overnight fast. Subsequently, the blood was incubated at room temperature for at least 30 min but no more than 2 h, and then centrifuged (1500 × g, 10 min, 4 °C). The resulting serum was transferred to cryovials and stored at − 80 °C in a freezer until further analysis.

### Hormone levels determination: leptin, ghrelin, and putative peptide YY

The serum hormone levels were assessed in duplicate following prior dilution using a commercially available enzyme-linked immunosorbent assay (ELISA) kit (Wuhan Fine Biotech Co., Ltd., Wuhan, China), as per the manufacturer's instructions. The limit of detection for each hormone was determined as follows: leptin, 18.75 pg/ml; ghrelin, 1.125 pg/ml; putative peptide YY-3, 3.75 pg/ml. The within-assay and between-assay coefficients of variation were both found to be below 8% and 10%, respectively. Clinical parameters were retrieved from patient medical records, while blood morphology was analyzed using a hematology analyzer from Siemens Healthineers, Germany.

### Statistical analysis

All statistical analyses were conducted using the STATISTICA software package (version 13.3, StatSoft Inc. 2017, Tulsa, OK, USA). The data were presented as either mean and standard deviation or median with the range. Most variables did not adhere to a normal distribution, as confirmed by the Shapiro–Wilk test, necessitating the utilization of non-parametric tests. For comparisons between two independent groups, the Mann–Whitney U test was employed, while for multiple comparisons, the Kruskal–Wallis ANOVA was utilized. Correlation analysis was performed using the Spearman correlation test. A p-value of less than 0.05 was considered statistically significant.

## Results

The study included 62 patients with FASDs, comprising 31 boys and 31 girls. Simultaneously, 23 healthy children, consisting of 16 boys and 7 girls (30.5%), were enrolled in the study. Table 1 presents the basic anthropometric and clinical laboratory parameters of both FASD patients and healthy controls.

**Table 1:** Baseline demographic and clinical data of the study participants.

		FASD	Healthy controls	*p* value
Sex (F/M)		31/31	7/16	
Age (years)	Mean ± SD	7.52 ± 4.16	7.45 ± 5.12	0.847
	Range	0.42‒16.50	0.42‒17.00	
BMI percentile	Mean ± SD	32.38 ± 31.24	60.71 ± 27.03	**0.035**
	Range	0.1‒99.9	12.0‒99.0	
Clinical laboratory markers				
Cholesterol (mg/dL) Norm < 190	Median	150.00	155.0	0.971
	Range	76.0–244.0	126.0–191.0	
LDL (mg/dL) Norm < 135	Median	90.0	95.0	0.827
	Range	31.0–163.0	72.0–104.0	
HDL (mg/dL) Norm > 40	Median	53.0	53.0	0.856
	Range	24.0–108.0	42.0–59.0	
Triglycerides (mg/dL) Norm < 150	Median	74.0	65.0	0.753
	Range	30.0–241.0	38.0–141.0	
Glucose (mg/dL) Norm (70–99)	Median	84.0	87.0	0.669
	Range	72.0–99.0	68.0–94.0	
Insulin (mIU/ml) Norm < 15	Median	4.85	2.05	0.167
	Range	1.25–17.0	1.0–9.03	
HbA1c (%) Normal range (4–6)	Median	5.36	5.41	0.774
	Range	4.71–5.86	5.26–5.55	
HOMA-IR Norm < 2.5	Median	0.96	–	–
	Range	0.23–3.62	–	
Cortisol (µg/dl)	Median	8.80	11.80	0.139
	Range	3.5–26.0	6.3–19.1	

BMI percentile: body mass index percentile; LDL: low-density lipoprotein; HDL: high-density lipoprotein; HbA1c: glycated hemoglobin; HOMA-IR: homeostasis model assessment of insulin resistance, data are presented as median and range; differences between means were analyzed using Mann–Whitney U test.

Significant values are in bold.

With the exception of BMI percentile, none of the parameters under investigation exhibited statistically significant distinctions. There were no notable variations in age between patients diagnosed with FASD and those in the healthy control cohort. However, we did observe a significant age disparity within the FASD subgroups (refer to Table 2), largely due to the younger age of individuals in the FASD risk category. Nonetheless, the disparities between the FAS and ND-PAE groups did not reach statistical significance (*p* = 0.686). As mentioned above, the BMI percentile values were markedly lower in the FASD cohort compared to the control group (*p* = 0.035). Differences in BMI were associated with disease severity and the BMI percentile in the FAS subgroup was notably lower than that in the ND-PAE group.

**Table 2:** Baseline demographic and clinical data of the FASD subgroups.

		FAS	ND-PAE	FASD risk	*p* value
Sex (F/M)		14/12	15/16	2/3	
Age (years)	Mean ± SD	7.91 ± 4.77	8.13 ± 3.32	2.25 ± 1.26	**0.030**
	Range	0.42‒16.50	2.08‒13.50	1.17‒4.42	
BMI percentile	Mean ± SD	22.12 ± 27.51	42.04 ± 33.02	27.33 ± 13.87	**0.018**
	Range	0.10–78.0	0.1‒99.9	12.0‒39.0	
Clinical laboratory markers					
Cholesterol (mg/dL) Norm < 190	Median	154.50	161.0	141.0	0.513
	Range	76.0–238.0	114–244	104.0–185.0	
LDL (mg/dL) Norm < 135	Median	86.0	75.0	84.0	0.854
	Range	31.0–143.0	114–244	33.0–119.0	
HDL (mg/dL) Norm > 40	Median	49.50	53.0	46.0	0.715
	Range	33.0–80.0	24–108	33.0–71.0	
Triglycerides (mg/dL) Norm < 150	Median	64.0	75.0	75.0	0.937
	Range	30.0–229.0	34–241	55.0–99.0	
Glucose (mg/dL) Norm (70–99)	Median	82.0	87.0	80.0	0.409
	Range	72.0–99.0	74.0–99.0	76.0–91.0	
Insulin (mIU/ml) Norm < 15 mIU/ml	Median	5.10	4.22	3.22	0.558
	Range	1.41–16.46	1.56–13.97	1.25–17.0	
HbA1c (%) Normal range (4–6)	Median	5.24	5.45	5.35	0.039*
	Range	4.81–5.86	4.89–5.85	4.71–5.53	
HOMA-IR Norm < 2.5	Median	1.07	1.29	1.73	0.790*
	Range	0.27–3.62	0.31–3.53	0.23–3.36	
Cortisol (µg/dl)	Median	9.9	7.95	9.0	0.649
	Range	4.30‒18.9	3.5‒26.0	6.1‒23.2	

BMI percentile: body mass index percentile; LDL: low-density lipoprotein; HDL: high-density lipoprotein; HbA1c: glycated hemoglobin; HOMA-IR: homeostasis model assessment of insulin resistance, data are presented as median and range; comparison between means were analyzed using Kruskal–Wallis test or “*”Mann–Whitney U test (comparison between FAS and ND-PAE only).

Significant values are in bold.

In the FAS subgroup, 42.31% of children exhibited low height (< 3rd percentile), with the majority being girls (90.91%) compared to boys (9.09%). Conversely, within the ND-PAE subgroup, 18.18% of children had low height, with the largest segment falling between the 25th and 50th percentiles (27.27%), evenly distributed between girls and boys (50% each). In the FASD RISK group, 20% of children displayed low growth. No statistically significant differences were noted between the groups in terms of biochemical parameters such as lipid profile, glucose, insulin, or HOMA-IR.

**Figure 1:**
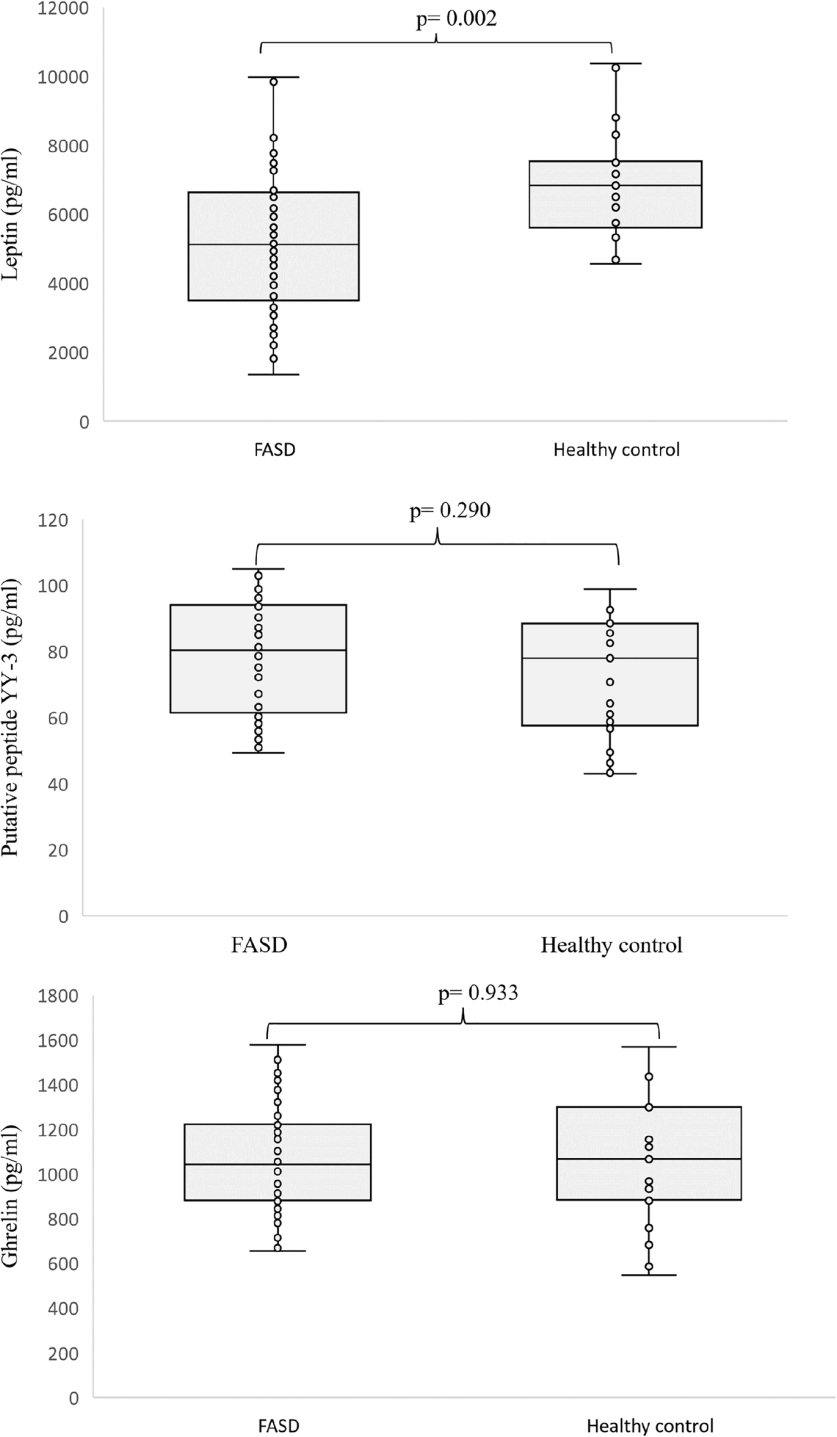
Level of Leptin, Ghrelin, and Putative peptide YY-3 in patients with FASD as compared to healthy participants.

The hormone levels examined are illustrated in Fig. 1. A notable reduction in leptin levels was observed in the serum of individuals with FASD compared to healthy counterparts (*p* = 0.002). Significant differences in leptin levels were also observed (Table 3). However, caution is warranted in interpreting these results due to the potential confounding effect of younger age observed in FASD risk patients, which may impact leptin secretion. Specifically comparing the FAS and ND-PAE groups revealed no disparities (*p* = 0.732). Concentrations of other studied hormones, such as PYY and ghrelin, demonstrated similarity between individuals with FASD and controls, as well as within FASD subgroups. Table 4 provides a comparison of hormone concentrations between females and males with FASD, indicating no discernible differences in leptin, ghrelin, and PYY levels between sexes in the FASD cohort.

**Table 3:** Leptin, ghrelin and putative peptide YY-3 levels in subgroups of patients with FASD.

		FAS	ND-PAE	FASD risk	*p* value
Leptin (pg/ml)	Median	5021.84	5347.11	4304.21	**0.017**
	Range	1359.47–7930.53	2713.16–9970.53	2532.63–7501.58	
Ghrelin (pg/ml)	Median	983.68	1061.38	1172.28	0.893
	Range	669.35–1578.26	655.77–1987.57	784.41–1511.68	
Putative peptide YY-3 (pg/ml)	Median	80.82	75.07	81.18	0.588
	Range	54.01–103.62	49.21–104.93	54.21–97.57	

Data are presented as median and range; comparison between means were analyzed using Kruskal- Wallis test.

Significant values are in bold.

**Table 4:** Hormone levels by sex of FASD patients.

Hormone	FASD			
		Girls	Boys	*p* value
Leptin (pg/ml)	Median	5292.11	4354.21	0.23
	Range	1359.47–9970.53	1812.63–7930.53	
Ghrelin (pg/ml)	Median	1053.87	1028.3	0.951
	Range	655.77–1987.57	714.42–1511.68	
Putative peptide YY-3 (pg/ml)	Median	80.79	76.32	0.855
	Range	50.92–104.93	49.21–104.8	

Data are presented as median and range; comparison between means were analyzed by Mann–Whitney U test.

The next stage of data analysis involved evaluating the correlations between the hormones levels of the studied and the clinical parameters of patients with FASD (Table 5). Leptin showed a positive correlation with blood glucose concentration (r_s_ = 0.289, *p* = 0.028) and a negative correlation with cortisol levels (r_s_ = − 0.307, *p* = 0.018). Putative peptide YY-3 and ghrelin were not correlated with any clinical parameter. Furthermore, we examined the correlations between the hormones tested and other appetite-regulating hormones, including proopiomelanocortin (POMC), Agouti-signaling protein (ASP), kisspeptin (KISS1), neuropeptide Y (NPY) and alpha-melanocyte-stimulating hormone (α-MSH), which we previously determined by us^25,26^. We found weak correlation with POMC: a negative for PYY (r_s_ = − 0.289, *p* = 0.024) and positive for ghrelin (r_s_ = 0.268, *p* = 0.042). Leptin was also positively associated with the levels of POMC (r_s_ = 0.339, *p* = 0.007) and ASP (r_s_ = 0.31, *p* = 0.014).

**Table 5:** Spearman’s rank correlation coefficients (r_s_) and p values between hormone concentrations and clinical features of studied patients.

		Age	Cor	Ch	LDL	HDL	TGL	Glu	Ins	HOMA-IR	HbA1c	BMI per	Leptin	Ghrelin	POMC	ASP	PYY	KISS1	NPY	α-MSH
Leptin	R	0.058	− 0.307	0.011	0.053	− 0.176	0.002	0.289	0.163	0.191	0.011	0.225		0.226	0.339	0.31	− 0.1	0.095	0.051	− 0.171
	p	0.652	**0.018**	0.931	0.684	0.174	0.988	**0.028**	0.239	0.166	0.935	0.090		0.088	**0.007**	**0.014**	0.443	0.462	0.694	0.189
Ghrelin	R	− 0.005	− 0.188	0.085	0.099	− 0.031	0.036	0.176	0.037	0.061	− 0.218	0.119	0.226		0.268	0.119	− 0.041	0.129	0.050	0.041
	p	0.971	0.170	0.531	0.462	0.822	0.793	0.195	0.797	0.669	0.128	0.393	0.088		**0.042**	0.375	0.761	0.334	0.707	0.761
Putative peptide YY-3	R	− 0.129	0.170	− 0.164	− 0.171	− 0.168	− 0.027	− 0.226	− 0.23	− 0.226	− 0.097	0.048	− 0.1	− 0.041	− 0.289	− 0.222		0.044	0.109	0.250
	p	0.321	0.202	0.21	0.192	0.2	0.84	0.091	0.097	0.104	0.49	0.725	0.443	0.761	**0.024**	0.086		0.738	0.405	0.054

Cor: cortisol; Ch: cholesterol; LDL: low density lipoprotein; HDL: high density lipoprotein; TGL: triglycerides; Glu: glucose, Ins-insulin; HOMA-IR: homeostasis model assessment of insulin resistance; HbA1c: glycated hemoglobin; BMI per: body mass index percentile; POMC: proopiomelanocortin; ASP: Agouti-signaling protein; PYY: Putative peptide YY-3; KISS1: kisspeptin; NPY: neuropeptide Y; α-MSH: alpha-melanocyte-stimulating hormone.

Significant values are in bold.

## Discussion

This study has investigated the effects of prenatal exposure to ethanol on serum levels of hormones that regulate appetite: leptin, ghrelin and putative peptide YY-3. The main finding of our study is a significantly decreased leptin level in FASD compered to healthy control. We have also observed correlations of leptin levels with glucose, POMC, ASP and cortisol. No significant differences were found in PYY and ghrelin concentrations between FASD subgroups as well as controls. Sex did not affect the levels of hormones studied.

Leptin, an anorexigenic hormone discovered in 1994, is primarily secreted from white adipose tissue. It acts through the activation of transmembrane leptin receptors, which are widely distributed in peripheral organs as well as the central nervous system^27^. Feeding or obesity increases circulating leptin levels, while fasting decreases them^28^. Besides regulating food intake and body mass, this hormone also influences reproductive functioning and plays a vital role in fetal growth, proinflammatory immune responses, lipolysis, and angiogenesis^21,29,30^. Its expression and secretion are regulated by many factors, including inflammatory cytokines, insulin, and glucocorticoids^31^. Recent studies have underlined the impact of alcohol consumption on increased short-term energy intake due to appetite stimulation, leading to weight gain and obesity^32,33^.

Alcohol exerts an inhibitory effect on leptin secretion, although the data are not consistent^34^. Alcohol can also affect the leptin receptor. In mice consuming ethanol for 5 weeks, the overall expression of leptin receptors was elevated, whereas the expression of the functionally active long form of leptin receptor decreased in the hypothalamus and perigonadal fat^35^. In humans, soluble leptin receptor levels in alcohol-dependent patients were higher than in the control group and decreased during the abstinence program^36^. Based on these studies, it can be assumed that improper development in children with FASD may be associated with alterations in leptin receptor expression. Furthermore, leptin indirectly regulates the hypothalamic–pituitary–adrenal axis and the cortisol-mediated stress response, possibly influencing alcohol craving and withdrawal^37^. There is growing evidence that appetite-regulating peptides such as leptin and ghrelin are altered in alcoholism. The brain POMC system plays critical roles not only in regulating energy homeostasis but also in modulating alcohol intake^38^. The POMC system, which is susceptible to ethanol influence, is also intricately linked with leptin regulation. Ethanol exposure harms POMC neurons and decreases its secretion^25,39,40^. Furthermore, some evidence indicates that leptin can regulate human adrenal function through its receptors in the hypothalamus, where it modulates the secretion of corticotropin-releasing hormone and, consequently, ACTH in the pituitary^41^. Leptin inhibits ACTH-stimulated steroid release by all three zones within the adrenal glands, with the most severe reduction in cortisol production^42^. This may be a protective mechanism opposite to the local situation in visceral adipose tissue, where glucocorticoid receptors and 11β-hydroxysteroid dehydrogenase type 1, which converts inactive cortisone to active cortisol, become upregulated in obesity^43^. These findings are supported in our study, by the results of correlation analysis, which reveal negative correlations between leptin and cortisol levels, as well as positive correlations between leptin and POMC. However, contrary to many previous studies, we did not find any association between the concentration of circulating leptin and the BMI of patients^21,44–46^. Although, as in our study, several reports also found no correlation between BMI and leptin levels^47^. Higher serum leptin levels have been shown to correlate with lower BMI in childhood and a reduced predisposition to developing metabolic disorders in adolescence and adulthood^48^. The effect of leptin on glucose metabolism and insulin secretion has been the subject of numerous studies^49,50^. We have found a weak positive correlation between leptin and glucose levels, but we have not observed any relationship between leptin concentrations and insulin, as well as insulin resistance indicators such as HOMA-IR, as previously described. Similar to previous studies, we found higher insulin concentrations in girls compared to boys, although the difference was not statistically significant^51,52^.

To date, there is a lack of knowledge about the effect of alcohol consumption during pregnancy on the secretion of appetite-regulating hormones, such as leptin or ghrelin, during infancy and childhood. Only a limited number of studies have addressed this issue. We have found significant differences in leptin levels among subjects with FASD compared to controls, as well as among FASD subgroups. The disease severity appears to negatively impact leptin secretion, except for the FASD risk group, which could be attributed to the significantly younger age of the participants. Children exposed to alcohol prenatally may display pre- and postnatal growth retardation, a defining characteristic of FASD^53^. Lower serum leptin levels were observed during the first 2 years of life in children exposed to ethanol^54^, which was also confirmed in our FASD risk subgroup, with median age 2.25 ± 1.26. Similar results were observed in animal model, where prenatal alcohol exposure led to reduced leptin levels in the subcutaneous adipose tissue of neonatal rats but not in adult rats^55^.

Ghrelin, the "hunger hormone," primarily originates from enteroendocrine cells in the stomach and regulates food intake by signaling hunger during fasting. It exhibits antagonistic action to leptin and its levels decrease in obesity due to positive energy balance^56–58^. Besides its presence in the arcuate nucleus of the hypothalamus, ghrelin receptors in the mesolimbic dopaminergic system, suggest its role in regulating rewards in substance use disorders such as alcohol addiction, primarily through activating the cholinergic-dopaminergic reward link^59^. Animal studies have shown the beneficial effects of ghrelin receptor antagonists in reducing the intake of palatable foods, suppressing preferences for caloric foods, and decreasing alcohol intake, as well as suppressing alcohol-induced reward^60^. Additionally, ghrelin demonstrates potent central gastroprotective activity against ethanol-induced lesions in animal models^61^. While the specific role of ghrelin in FASD remains unclear, studies in rodents suggest that prenatal alcohol exposure can alter ghrelin levels in offspring, potentially affecting appetite regulation and increasing the risk of obesity later in life^62–64^. We found a positive correlation between ghrelin and POMC, involved in energy balance and neuroendocrine function^65^. Early exposure to alcohol during pregnancy has been shown to alter the activity of the hypothalamic–pituitary–adrenal axis, increasing the risk of anxiety, depression, and alcohol-related disorders^25,66,67^. Contrary to previous findings, we did not observe a relationship between ghrelin levels and BMI or glucose levels^46,68^. The correlation analysis between hormones regulating appetite also indicated no association between leptin and ghrelin, which differs with many previous studies^57,69^. Our results showed no change in circulating ghrelin levels between the study groups; therefore, we can assume that the ghrelin mediation system is not disturbed by prenatal alcohol exposure.

Putative peptide YY-3, a gut-derived hormone, is recognized for its role in reducing short-term food intake by stimulating hypothalamic neuropeptide Y receptors (56). PYY is released from enteroendocrine cells, primarily found in the distal gastrointestinal tract postprandially in proportion to the ingested calories, reaching its maximum levels after 1–2 h^70^. The anorexigenic effect is likely induced by stimulation of the Y2 receptor in the arcuate nucleus, as feeding is abrogated in Y2 receptor knockout mice^71^. PYY slows gastric emptying and gastrointestinal motility, inhibits gastric acid secretion, gallbladder contractions, and secretion of pancreatic exocrine enzymes^72^. Several studies have investigated the association of PYY secretion with various clinical and nutritional parameters such as age, sex, BMI, adiposity, dietary fats, or acute exercise^72–75^. We did not find significant changes in PYY levels between participants with FASD and healthy individuals, nor within subgroups of FASD. The influence of fetal alcohol exposure on PYY secretion remains uncharacterized. Higher plasma levels of PYY have been reported in patients with anorexia nervosa compared to obese individuals, or morbidly obese as well as lean individuals^76^ but no differences were also reported^77^. Significantly lower PYY concentrations have been described in children born small for gestational age, a common clinical features of FAS^78^. The effect of alcohol consumption was investigated by Calissendorff and colleagues^79^. They reported that unlike ghrelin, alcohol consumption does not affect changes in PYY levels. PYY levels have shown no significant changes after acute and chronic ethanol administration in animal models^80^. However, previous study has indicated stimulatory effect of PYY on POMC neurons^81^, which are damaged in fetus by prenatal alcohol exposure^40^. Thus, assessing the definitive effect of this exposure on nutrition regulation is challenging. Correlation analysis revealed only week negative association of PYY with POMC, and very close to statistically significant positive correlation with α-MSH (R = 0.250, *p* = 0.054). As mentioned before, previous studies have reported stimulatory effect of PYY on POMC expression and inhibitory effect on NPY/AgRP neurons, but these findings were not supported in our study^81^. In the correlation analysis of many appetite-regulating hormones, only a few were related, such as leptin and POMC, ASP and cortisol, ghrelin and POMC, PYY and POMC, which may suggest that food intake and hunger regulation is a complex and multifactorial process in which there is no direct activation/secretion of one hormone after the release of another, even if they act in opposing ways.

Although our study offers new insights into the hormonal regulation of food intake and energy balance in children prenatally exposed to alcohol, we acknowledge several limitations. The number of participants may not have been sufficient to definitively determine the effect of prenatal alcohol exposure on the subsequent secretion of leptin, ghrelin and PYY. The size of the subgroups, especially the FASD risk group, was relatively low, which could have affected the reliability of the statistical analysis. We also did not assess the intensity of physical activity in the study population, although it is known that exercise can alter the levels of ghrelin and PYY. Finally, the limited number of scientific studies describing the hormonal regulation of food intake in FASD complicates the interpretation of the results.

## Conclusions

We found lower serum levels of leptin in patients with FASD compared to healthy subjects, while there was no difference in the concentration of ghrelin and PYY. The levels of hormones studied were not affected by sex or membership in FASD subgroups, except for leptin. We also observed some interesting correlations between the levels of the hormones studied and the parameters describing the clinical condition of the patients, including the association of leptin levels with glucose, POMC, ASP, and cortisol, as well as ghrelin and PYY with POMC. Basic anthropometric and clinical parameters such as age, BMI, or lipid profile did not correlate with any of the investigated hormones. However, the observed alterations in the rate of development among individuals with FASD may stem from disrupted appetite control. These findings should be considered in the appropriate and holistic management of patients.

## Data Availability

The data used and/or analyzed during the current study available from the corresponding author on reasonable request.

## References

[1] Wozniak JR, Riley EP, Charness ME (2019). Clinical presentation, diagnosis, and management of fetal alcohol spectrum disorder. Lancet Neurol..

[2] Moghetti P (2000). Comparison of spironolactone, flutamide, and finasteride efficacy in the treatment of hirsutism: A randomized, double blind, placebo-controlled Trial1. J. Clin. Endocrinol. Metab..

[3] Hellemans KGC, Sliwowska JH, Verma P, Weinberg J (2010). Prenatal alcohol exposure: Fetal programming and later life vulnerability to stress, depression and anxiety disorders. Neurosci. Biobehav. Rev..

[4] Hoyme HE (2016). Updated clinical guidelines for diagnosing fetal alcohol spectrum disorders. Pediatrics.

[5] Astley SJ (2013). Validation of the fetal alcohol spectrum disorder (FASD) 4-digit diagnostic code. J. Popul. Ther. Clin. Pharmacol..

[6] Cook JL (2016). Fetal alcohol spectrum disorder: A guideline for diagnosis across the lifespan. CMAJ.

[7] Bertrand J, Floyd RL, Weber MK (2005). Guidelines for Identifying and referring persons with fetal alcohol syndrome. Morb. Mortal. Wkly. Rep. Recomm. Rep..

[8] Jones KL, Smith DW, Ulleland CN, Streissguth P (1973). Pattern of malformation in offspring of chronic alcoholic mothers. Lancet Lond. Engl..

[9] Popova S, Lange S, Probst C, Gmel G, Rehm J (2017). Estimation of national, regional, and global prevalence of alcohol use during pregnancy and fetal alcohol syndrome: A systematic review and meta-analysis. Lancet Glob. Health.

[10] Dejong K, Olyaei A, Lo JO (2019). Alcohol use in pregnancy. Clin. Obstet. Gynecol..

[11] Carter RC (2016). Fetal alcohol growth restriction and cognitive impairment. Pediatrics.

[12] Lange S (2017). Global prevalence of fetal alcohol spectrum disorder among children and youth: A systematic review and meta-analysis. JAMA Pediatr..

[13] May PA (2014). Prevalence and characteristics of fetal alcohol spectrum disorders. Pediatrics.

[14] May PA (2013). Approaching the prevalence of the full spectrum of fetal alcohol spectrum disorders in a South African population-based study. Alcohol. Clin. Exp. Res..

[15] May PA (2006). Epidemiology of FASD in a province in Italy: Prevalence and characteristics of children in a random sample of schools. Alcohol. Clin. Exp. Res..

[16] Oei JL (2020). Alcohol use in pregnancy and its impact on the mother and child. Addiction.

[17] Werts RL, Van Calcar SC, Wargowski DS, Smith SM (2014). Inappropriate feeding behaviors and dietary intakes in children with fetal alcohol spectrum disorder or probable prenatal alcohol exposure. Alcohol. Clin. Exp. Res..

[18] Klug MG, Burd L, Martsolf JT, Ebertowski M (2003). Body mass index in fetal alcohol syndrome. Neurotoxicol. Teratol..

[19] Amos-Kroohs RM (2016). Abnormal eating behaviors are common in children with fetal alcohol spectrum disorder. J. Pediatr..

[20] Druce M (2005). The regulation of appetite. Arch. Dis. Child..

[21] Obradovic M (2021). Leptin and obesity: Role and clinical implication. Front. Endocrinol..

[22] Reidelberger R, Haver A, Chelikani PK (2013). Role of peptide YY(3–36) in the satiety produced by gastric delivery of macronutrients in rats. Am. J. Physiol. - Endocrinol. Metab..

[23] Karra E, Chandarana K, Batterham RL (2009). The role of peptide YY in appetite regulation and obesity. J. Physiol..

[24] Okulicz-Kozaryn K, Maryniak A, Borkowska M, Śmigiel R, Dylag KA (2021). Diagnosis of fetal alcohol spectrum disorders (FASDs): Guidelines of interdisciplinary group of polish professionals. Int. J. Environ. Res. Public. Health.

[25] Podgórski R, Galiniak S, Mazur A, Domin A (2023). The association of the hypothalamic-pituitary-adrenal axis with appetite regulation in children with fetal alcohol spectrum disorders (FASDs). Nutrients.

[26] Podgórski R, Galiniak S, Mazur A, Podgórska D, Domin A (2023). Serum levels of hormones regulating appetite in patients with fetal alcohol spectrum disorders. Nutrients.

[27] Münzberg H, Morrison CD (2015). Structure, production and signaling of leptin. Metabolism.

[28] Ahima RS (1996). Role of leptin in the neuroendocrine response to fasting. Nature.

[29] Farr OM, Gavrieli A, Mantzoros CS (2015). Leptin applications in 2015: What have we learned about leptin and obesity?. Curr. Opin. Endocrinol. Diabetes Obes..

[30] De Vos P, Saladin R, Auwerx J, Staels B (1995). Induction of ob gene expression by corticosteroids is accompanied by body weight loss and reduced food intake. J. Biol. Chem..

[31] Dagogo-Jack S (2001). Human leptin regulation and promise in pharmacotherapy. Curr. Drug Targets.

[32] Caton SJ, Nolan LJ, Hetherington MM (2015). Alcohol, appetite and loss of restraint. Curr. Obes. Rep..

[33] Yeomans MR (2004). Effects of alcohol on food and energy intake in human subjects: evidence for passive and active over-consumption of energy. Br. J. Nutr..

[34] Yeomans MR, Caton S, Hetherington MM (2003). Alcohol and food intake. Curr. Opin. Clin. Nutr. Metab. Care.

[35] Obradovic T, Meadows GG (2002). Chronic ethanol consumption increases plasma leptin levels and alters leptin receptors in the hypothalamus and the perigonadal fat of C57BL/6 mice. Alcohol. Clin. Exp. Res..

[36] Weinland C, Tanovska P, Kornhuber J, Mühle C, Lenz B (2020). Serum lipids, leptin, and soluble leptin receptor in alcohol dependence: A cross-sectional and longitudinal study. Drug Alcohol Depend..

[37] Kiefer F, Wiedemann K (2004). Neuroendocrine pathways of addictive behaviour. Addict. Biol..

[38] Wurst FM (2007). Alcoholism, craving, and hormones: The role of leptin, ghrelin, prolactin, and the pro-opiomelanocortin system in modulating ethanol intake. Alcohol. Clin. Exp. Res..

[39] Mukherjee S (2020). Alcohol increases exosome release from microglia to promote complement C1q-induced cellular death of proopiomelanocortin neurons in the hypothalamus in a rat model of fetal alcohol spectrum disorders. J. Neurosci..

[40] Bekdash R, Zhang C, Sarkar D (2014). Fetal alcohol programming of hypothalamic proopiomelanocortin system by epigenetic mechanisms and later life vulnerability to stress. Alcohol. Clin. Exp. Res..

[41] Malendowicz, L. K., Rucinski, M., Belloni, A. S., Ziolkowska, A. & Nussdorfer, G. G. Leptin and the Regulation of the Hypothalamic–Pituitary–Adrenal Axis. in *International Review of Cytology* vol. 263 63–102 (Elsevier, 2007) 10.1016/S0074-7696(07)63002-2 17725965

[42] Glasow A, Bornstein SR (2000). Leptin and the adrenal gland. Eur. J. Clin. Invest..

[43] Walker GE, Marzullo P, Prodam F, Bona G, Di Blasio AM (2014). Obesity modifies expression profiles of metabolic markers in superficial and deep subcutaneous abdominal adipose tissue depots. Endocrine.

[44] Savino F (2016). Mother and infant body mass index, breast milk leptin and their serum leptin values. Nutrients.

[45] Khan Z (2019). Correlation between serum leptin level and body mass index (BMI) in patients with type II diabetes mellitus. J. Pak. Med. Assoc..

[46] Ikezaki A (2002). Fasting plasma ghrelin levels are negatively correlated with insulin resistance and PAI-1, but not with leptin, in obese children and adolescents. Diabetes.

[47] Mohanraj J, D’Souza UJA, Fong SY, Karkada IR, Jaiprakash H (2022). Association between Leptin (G2548A) and leptin receptor (Q223R) polymorphisms with plasma leptin, BMI, stress, sleep and eating patterns among the multiethnic young Malaysian adult population from a healthcare university. Int. J. Environ. Res. Public. Health.

[48] Savino F (2013). High serum leptin levels in infancy can potentially predict obesity in childhood, especially in formula-fed infants. Acta Paediatr. Oslo Nor..

[49] Fischer S (2002). Insulin-resistant patients with type 2 diabetes mellitus have higher serum leptin levels independently of body fat mass. Acta Diabetol..

[50] Pereira S, Cline DL, Glavas MM, Covey SD, Kieffer TJ (2021). Tissue-specific effects of leptin on glucose and lipid metabolism. Endocr. Rev..

[51] Licinio J (1998). Sex differences in circulating human leptin pulse amplitude: clinical implications. J. Clin. Endocrinol. Metab..

[52] Haleem DJ, Sheikh S, Fawad A, Haleem MA (2017). Fasting leptin and glucose in normal weight, over weight and obese men and women diabetes patients with and without clinical depression. Metab. Brain Dis..

[53] Caputo C, Wood E, Jabbour L (2016). Impact of fetal alcohol exposure on body systems: A systematic review: Impact of Fetal Alcohol Exposure on Body Systems. Birth Defects Res. Part C Embryo Today Rev..

[54] Aros S (2011). Effects of prenatal ethanol exposure on postnatal growth and the insulin-like growth factor axis. Horm. Res. Paediatr..

[55] Chen L, Nyomba BLG (2003). Effects of prenatal alcohol exposure on glucose tolerance in the rat offspring. Metabolism..

[56] Makris MC (2017). Ghrelin and obesity: Identifying gaps and dispelling myths. A reappraisal. Vivo Athens Greece.

[57] Tschöp M (2001). Circulating ghrelin levels are decreased in human obesity. Diabetes.

[58] Scerif M, Goldstone AP, Korbonits M (2011). Ghrelin in obesity and endocrine diseases. Mol. Cell. Endocrinol..

[59] Koopmann A, Schuster R, Kiefer F (2018). The impact of the appetite-regulating, orexigenic peptide ghrelin on alcohol use disorders: A systematic review of preclinical and clinical data. Biol. Psychol..

[60] Mokrosiński J, Holst B (2010). Modulation of the constitutive activity of the ghrelin receptor by use of pharmacological tools and mutagenesis. Methods Enzymol..

[61] Sibilia V (2003). Ghrelin protects against ethanol-induced gastric ulcers in rats: Studies on the mechanisms of action. Endocrinology.

[62] Duart-Castells L (2021). Effects of high-fat diet and maternal binge-like alcohol consumption and their influence on cocaine response in female mice offspring. Int. J. Neuropsychopharmacol..

[63] Larsson A, Edström L, Svensson L, Söderpalm B, Engel JA (2005). Voluntary ethanol intake increases extracellular acetylcholine levels in the ventral tegmental area in the rat. Alcohol Alcohol..

[64] Jerlhag E (2009). Requirement of central ghrelin signaling for alcohol reward. Proc. Natl. Acad. Sci. U. S. A..

[65] Gallo-Payet N (2016). 60 years of POMC: Adrenal and extra-adrenal functions of ACTH. J. Mol. Endocrinol..

[66] Wieczorek L, Fish EW, O’Leary-Moore SK, Parnell SE, Sulik KK (2015). Hypothalamic-pituitary-adrenal axis and behavioral dysfunction following early binge-like prenatal alcohol exposure in mice. Alcohol.

[67] Govorko D, Bekdash RA, Zhang C, Sarkar DK (2012). Male germline transmits fetal alcohol adverse effect on hypothalamic proopiomelanocortin gene across generations. Biol. Psychiatry.

[68] Zhang DL (2018). Cord blood insulin, IGF-I, IGF-II, leptin, adiponectin and ghrelin, and their associations with insulin sensitivity, β-cell function and adiposity in infancy. Diabet. Med..

[69] Stylianou C, Galli-Tsinopoulou A, Koliakos G, Fotoulaki M, Nousia-Arvanitakis S (2007). Ghrelin and leptin levels in young adults with cystic fibrosis: Relationship with body fat. J. Cyst. Fibros. Off. J. Eur. Cyst. Fibros. Soc..

[70] Batterham RL (2002). Gut hormone PYY3-36 physiologically inhibits food intake. Nature.

[71] Batterham RL, Bloom SR (2003). The gut hormone peptide YY regulates appetite. Ann. N. Y. Acad. Sci..

[72] Cooper JA (2014). Factors affecting circulating levels of peptide YY in humans: A comprehensive review. Nutr. Res. Rev..

[73] Remmel L, Tillmann V, Purge P, Lätt E, Jürimäe J (2017). Associations of serum leptin, ghrelin and peptide YY levels with physical activity and cardiorespiratory fitness in adolescent boys with different BMI values. Biol. Sport.

[74] Batterham RL (2003). Inhibition of food intake in obese subjects by peptide YY3-36. N. Engl. J. Med..

[75] Kawano H (2013). Effects of different modes of exercise on appetite and appetite-regulating hormones. Appetite.

[76] Pfluger PT (2007). Effect of human body weight changes on circulating levels of peptide YY and peptide YY3–36. J. Clin. Endocrinol. Metab..

[77] Tam FI (2020). Peptide YY3–36 concentration in acute- and long-term recovered anorexia nervosa. Eur. J. Nutr..

[78] Wang L (2023). Relationship of glucagon-like peptide 1 and peptide YY with catch-up growth in children born small for gestational age. J. Clin. Res. Pediatr. Endocrinol..

[79] Calissendorff J, Danielsson O, Brismar K, Röjdmark S (2006). Alcohol ingestion does not affect serum levels of peptide YY but decreases both total and octanoylated ghrelin levels in healthy subjects. Metabolism.

[80] Simanowski UA (1989). Effects of acute and chronic ethanol administration on the gastrointestinal hormones gastrin, enteroglucagon, pancreatic glucagon and peptide YY in the rat. Digestion.

[81] Challis BG (2003). Acute effects of PYY3-36 on food intake and hypothalamic neuropeptide expression in the mouse. Biochem. Biophys. Res. Commun..

